# Central serous chorioretinopathy in active endogenous Cushing’s syndrome

**DOI:** 10.1038/s41598-021-82536-2

**Published:** 2021-02-02

**Authors:** Joost Brinks, Femke M. van Haalen, Thomas J. van Rijssen, Nienke R. Biermasz, Onno C. Meijer, Alberto M. Pereira, Camiel J. F. Boon, Elon H. C. van Dijk

**Affiliations:** 1 Department of Ophthalmology, Leiden University Medical Center, Department J3-S, P.O. Box 9600, 2300 RC Leiden, The Netherlands; 2 Department of Medicine, Division of Endocrinology, and Center for Endocrine Tumors, Leiden University Medical Center, Leiden, The Netherlands; 3 Department of Ophthalmology, Academic Medical Centers, University of Amsterdam, Amsterdam, The Netherlands

**Keywords:** Endocrinology, Medical research

## Abstract

Multiple case series have provided evidence for a relatively high incidence of central serous chorioretinopathy (CSC) in patients with active Cushing’s syndrome (CS). We describe the ophthalmological status in detail of consecutive patients with active endogenous CS (either de novo or recurrent active endogenous CS) in this prospective cohort study. All patients underwent complete ophthalmological examination, including multimodal imaging, which was performed shortly after establishing the diagnosis of active CS in hypercortisolemic state. Eleven CS patients (4 men, 7 women) with active hypercortisolism were included. Abnormalities reminiscent of (subclinical) CSC were found in 3 patients. Optical coherence tomography (OCT) revealed macular subretinal fluid in 1 patient, who was diagnosed as having active CSC and was successfully treated with half-dose photodynamic therapy. Two other patients showed CSC-like abnormalities: an unilateral pseudovitelliform lesion on OCT and hyperfluorescent changes on fluorescein angiography in one patient, and unilateral leakage on fluorescein angiography in the other patient. Mean subfoveal choroidal thickness on enhanced depth imaging OCT was 270 ± 40 μm (range, 178 – 357 μm). Retinal abnormalities resembling (subclinical) CSC may be more common than previously thought in patients with active CS, and may exist even in patients without visual complaints. Clinicians should have a low threshold for ophthalmological evaluation in case of a CS patient with visual symptoms since there may be therapeutic opportunities to prevent vision loss.

## Introduction

Cushing’s syndrome (CS) is a clinical entity that occurs after prolonged and excessive exposure to glucocorticoids. CS itself as well as comorbidities that are associated with CS result in decreased quality of life and an increased mortality risk^1,2^. CS is most often caused by exogenous use of glucocorticoids, whereas CS due to endogenous hypercortisolism is a rare condition with an estimated incidence of 1.2–2.4/million per year^2^. Patients with endogenous CS present with typical clinical findings, such as facial rounding (‘moon face’) and flushing (plethora), abdominal striae, muscle weakness, easy bruising, osteoporosis, hypertension, diabetes mellitus, and neuropsychiatric symptoms^3^. CS is most often dependent on pathological adrenocorticotropin (ACTH) secretion by a pituitary adenoma (‘Cushing’s disease’). However, CS can also be ACTH-independent in case of adrenal autonomous overproduction of cortisol, by an adrenal adenoma and seldom by an adrenal carcinoma^4^.

Visual complaints in CS can occur due to central serous chorioretinopathy (CSC), characterized by a (sub)acute accumulation of serous subretinal fluid (SRF) and detachment of the neuroretina. This is presumed to be secondary to damage to the retinal pigment epithelial (RPE) outer blood-retina barrier due to congestion, thickening, and hyperpermeability of the choroid^5–7^. Up to 5% of CS patients have been described to have had 1 or more episodes of CSC, which all occurred during a hypercortisolemic state^8^. As CSC can even be the principal manifestation of previously unrecognized, mildly symptomatic CS, ophthalmologists should have a high index of suspicion for systemic signs of CS that warrant referral to an endocrinologist^6^. Both exogenous glucocorticoid use, independent of the route of administration, and endogenous hypercortisolism have been described to be pronounced risk factors for CSC^6,9–13^. Although the exact pathogenetic mechanism of CSC is currently unclear, it has been hypothesized that hypercortisolism could increase the risk of developing CSC by altering capillary fragility, choroidal coagulation, systemic blood pressure, and/or fibroblastic activity^11,14^.

Choroidal hyperpermeability and thickening have been described to occur in both the affected and fellow eyes of CSC patients^5,15^. An increased choroidal thickness has been observed in patients with active endogenous CS, which has also been hypothesized to be a predisposing factor for CSC^16,17^. In a previous study, the assessment of the retina and choroid by means of optical coherence tomography (OCT) revealed retinal abnormalities within the CSC disease spectrum in 2 out of 11 patients^17^.

The diagnosis of abnormalities within the CSC spectrum can only be established with multimodal imaging including OCT. We conducted the first study in which ophthalmological imaging was performed in a consecutive series of patients with active endogenous CS without visual complaints, in order to evaluate the potential need of ophthalmological screening of any patient with CS.

## Methods

### Patient selection

Between August 2016 and November 2018, all consecutive patients diagnosed with CS on the outpatient clinic of the endocrinology department of our tertiary referral center were found eligible to participate in this study and referral to the ophthalmologist was discussed.

The diagnosis of CS was established based on clinical characteristics and corresponding abnormalities on biochemical evaluation in at least 2 out of 3 currently available screening tests, according to the Endocrine Society guideline^4^. Abnormal test results were defined as: insufficient suppression of cortisol secretion in the morning after 1 mg of dexamethasone the evening before (cortisol > 50 nmol/L), increased midnight saliva cortisol (> 5.7 nmol/L), increased 24-h urinary free cortisol excretion (> 150 nmol/L). Depending on the ACTH status of the patients, the etiology of CS was assessed either by magnetic resonance imaging scan of the brain or computed tomography scan of the adrenals/thorax. Eleven patients could be included in the current study, all with active (either as a first episode of CS or a new episode of CS in case of recurrence of disease), endogenous CS and without visual complaints at the moment of establishing the diagnosis. Five other patients met the inclusion criteria for this study, but could not be evaluated: 3 of these patients could not receive multimodal ophthalmological imaging before surgery due to logistical issues and the need for urgent pituitary surgery, and 2 did not prefer to participate.

The study adhered to the tenets of the Declaration of Helsinki. Both the institutional review board and the Medical Ethics Committee Leiden Den Haag Delft (METC-LDD) of Leiden University Medical Center approved this study (NL50816.058.14). Written informed consent was obtained from all participants.

### Ophthalmological evaluation

Complete ophthalmic examination started with an Early Treatment of Diabetic Retinopathy Study (ETDRS) best-corrected visual acuity (BCVA) measurement. Pupils were dilated by using 1% tropicamide and 5% phenylephrine, after which indirect ophthalmoscopy was performed. The obtained ophthalmological imaging consisted of digital color fundus photography (Topcon Corp., Tokyo, Japan), and OCT, enhanced depth imaging (EDI-)OCT of the choroid, fundus autofluorescence (FAF), and oral fluorescein angiography (FA), with a spectral-domain OCT device (Spectralis HRA + OCT; Heidelberg Engineering, Dublin, CA, United States). Subfoveal choroidal thickness (distance from the outer part of the hyperreflective RPE layer to the hyperreflective line of the inner surface of the sclera) was measured on EDI-OCT. Two experienced retina specialists (CJFB and EVD) evaluated the imaging. For oral FA images, 10 ml of 20% fluorescein was administered after a fasting period of at least 3 h, and photos were taken at 10, 15, 20, 25, and 30 min after ingestion.

When abnormal ophthalmological findings were present in patients with CS, patients were invited to the outpatient clinic for the ophthalmological assessment once again, after remission of CS. Moreover, when treatment and/or further follow-up of the ophthalmological situation was required according to the treating ophthalmologist, this was scheduled.

## Results

### Patient characteristics

Eleven patients (4 males, 7 females) with active CS were included in this study. The mean age of these patients was 52.6 ± 16.0 years (range, 22 – 75 years). CS was caused by Cushing’s disease (pituitary adenoma) in 7 patients, a unilateral adrenal adenoma in 2 patients, bilateral adrenal hyperplasia in 1 patient, and ACTH-dependent hypercortisolism of unknown origin (invisible pituitary adenoma or ectopic ACTH production of unknown origin) in 1 other patient. No other diseases or risk factors for which a possible association with CSC has been described, were present in the CS patients.

At the time of ophthalmological imaging, 8 patients were using antihypertensive drugs, for a mean duration of 8.3 ± 9.1 years (range, 0 – 25 years). One patient with persisting active CS with proven hypercortisolism on biochemical testing was still using hydrocortisone coverage in a low, physiological dosage because of concurrent insufficient cortisol peak production following dynamic testing. One other patient reported sporadic use of intranasal corticosteroids because of hay fever, and 1 other patient received an intra-articular shoulder injection with corticosteroids for the treatment of neuropathic pain 3 months prior to the study. None of the patients reported the use of either sildenafil or tadalafil, which has been associated with CSC^18^.

The mean duration between the diagnosis of active CS and ophthalmological phenotyping was 8 ± 6 weeks (range, 1 – 25 weeks). At the moment of ophthalmological phenotyping, 9 patients were already scheduled for surgery, but this had not been performed yet. Two other patients received non-surgical treatment, which had not started yet: this concerned 1 patient with a pituitary adenoma scheduled for levo-ketoconazol treatment in a trial setting and 1 patient with bilateral adrenal hyperplasia scheduled for leuporelin injections. Two patients had previously received pituitary surgery. In these patients, the active CS was considered to be a recurrence of the pituitary adenoma.

Furthermore, temporary preoperative treatment with oral medication for the inhibition of endogenous cortisol production was already started in 7 patients. Five patients were on temporary metyrapone treatment for a mean duration of 14 ± 13 days (range, 1 – 37 days) at the day of the visit to the department of Ophthalmology, and 1 other patient had received temporary ketoconazole treatment for 7 days. In another patient pasireotide treatment was started 3 days before ophthalmological evaluation. Biochemical evaluation showed a persistent hypercortisolemic state at the time of ophthalmological evaluation in all of these patients. Clinical characteristics of the patients are summarized in Table 1.

**Table 1:** Clinical characteristics of patients with active Cushing's syndrome.

Patient	Age	Sex	Presenting symptoms	Urinary free cortisol at diagnosis (xULN)	Type CS	Blood pressure (mmHg)	Received treatment at first visit outpatient clinic ophthalmology	Medication first visit outpatient clinic ophthalmology
1	63	M	Central adiposity, hematomas	2–3	Pituitary adenoma	152/87	-	Clopidogrel, pravastatin
2	58	F	Central adiposity, diabetes, hematomas, hirsutism, hypertension, muscle weakness	13–16	Pituitary adenoma	160/90	Metyrapone (3 days)	Atenolol, cotrimoxazol, digoxin, losartan, metformin, metyrapone, pantoprazole, phenprocoumon, potassium chloride, spironolactone
3	59	M	Central adiposity, diabetes, fatigue, hematomas, hypertension, psychosocial complaints	Cannot be interpreted due to medication use	Pituitary adenoma (recurrence of disease)	110/75	-	Calcium carbonate, carbasalate calcium, hydrocortisone, levothyroxine, metformin, metoprolol, oxazepam, paracetamol, pravastatin, testosterone
4	41	F	Central adiposity, fatigue, hematomas, hypertension, muscle weakness, psychosocial complaints	2–4	ACTH-dependent hypercortisolism with negative pituitary surgery	145/95	Metyrapone (1 day)	Labetalol, methyldopa, metyrapone, nifedipine, omeprazol, oxazepam
5	50	M	Central adiposity, hypertension, insomnia	2–6	Pituitary adenoma (recurrence of disease)	144/94	Pasireotide (3 days)	Levothyroxine, pasireotide, testosterone
6	66	F	Central adiposity, fatigue, hypertension, insomnia	1.5	Bilateral adrenal hyperplasia	150/100	-	Amlodipin, furosemide, levothyroxine, losartan, propranolol
7	22	F	Central adiposity, hypertension, oligomenorrhoe, psychosocial complaints	26	Pituitary adenoma	130/90	Metyrapone (37 days)	Amlodipin, cotrimoxazole, metyrapone, spironolactone
8	25	F	Amenorrhoe, central adiposity, fatigue, hematomas, psychosocial complaints	5–6	Pituitary adenoma	120/80	Metyrapone (19 days)	Metyrapone
9	75	F	Central adiposity, hematomas, insomnia	1.5–3.5	Pituitary adenoma	140/80	-	Alendronic acid, cholecalciferol, rivaroxaban
10	59	F	Central adiposity, fatigue, hypertension, muscle weakness, psychosocial complaints	2	Adrenal adenoma	165/87	Ketoconazole (7 days)	Amlodipin, cholecalciferol, gliclazide, hydrochlorothiazide, ketoconazole, levothyroxine, metformin, nebivolol, rosuvastatin
11	61	M	Hematomas, hypertension, muscle weakness, psychosocial complaints	2.5–3	Adrenal adenoma	150/90	Metyrapone (10 days)	Atorvastatin, cotrimoxazol, metformin, metoprolol, metyrapone, perindopril, spironolactone, tamsulosin

*ACTH* adrenocorticotropic hormoneadrenocorticotropic hormone, *CS* Cushing’s syndrome, *xULN* x upper limit of normal.

### Ophthalmological characteristics

An overview of the ophthalmological characteristics is shown in Table 2. Mean ETDRS BCVA of the 22 eyes was 82.3 ± 9.7 letters (range, 50 – 96 letters), with a mean spherical equivalent of the manifest refraction of 0.8 ± 3.3 diopters (range,-7.5 to + 7 diopters). Mean subfoveal choroidal thickness on EDI-OCT was 270 ± 40 μm (range, 178 – 357 μm), and no evident pachyvessels were seen in any of the patients.

**Table 2:** Ophthalmological characteristics of patients with active Cushing's syndrome.

Patient	Steroid use	Chorioretinal disease history	BCVA OD	BCVA OS	SPH EQ OD	SPH EQ OS	OCT OD	CT OD (µm)	OCT OS	CT OS (µm)	FAF OD	FAF OS	FA OD	FA OS
1*	–	–	94	90	0	+ 0.50	Parafoveal pseudovitelliform lesions, RPE alterations	301	–	317	HAF changes	–	HF lesions	–
2*	–	–	82	82	+ 1.00	+ 1.25	–	271	–	178	–	–	–	–
3*	HC (2012—current)	Central retinal vein occlusion OD (2012), grade 2 hypertensive retinopathy ODS (2015)	91	91	+ 1.25	+ 1.00	–	215	RPE alterations	257	–	–	Subtle HF lesions	Subtle HF lesions
4*	–	–	74	80	−7.50	−6.50	–	232	–	286	–	–	–	–
5	HC (1994—4 months before visit)	Epiretinal membrane, myelinated fibers ODS, congenital hypertrophy of RPE OD	50	83	+ 7.00	+ 5.50	Epiretinal membrane	–	Epiretinal membrane	296	Myelinated fibers	Myelinated fibers	CHRPE, myelinated fibers	Myelinated fibers
6*	–	Grade 4 hypertensive retinopathy ODS (2017)	75	78	+ 5.75	+ 5.00	SRF, flat irregular RPE detachment with midreflective accumulation beneath it, outer retinal changes, RPE alterations	357	–	335	HAF changes	–	Two parafoveal 'hot spots' of leakage	–
7	–	–	93	96	+ 0.50	−0.25	–	242	–	257	–	–	–	–
8	Intranasal (2017—current)	–	88	88	0	+ 0.25	–	259	–	242	–	–	–	–
9	–	Dry age-related macular degeneration	74	78	+ 2.25	+ 2.00	Age-related drusen	263	Age-related drusen	257	–	–	–	–
10	Intra-articular injection (3 months before visit)	–	85	82	+ 1.00	+ 1.00	–	299	–	277	–	–	–	–
11	–	–	73	83	−1.75	−1.50	–	250	–	291	HAF area	–	Single 'hot spot' of leakage	–

* = 2 or more visits. *BCVA* best-corrected visual acuity, *CHRPE* congenital hypertrophy of the retinal pigment epithelium, *CRVO* central retinal vein occlusion, *CT* subfoveal choroidal thickness, *FA* fluorescein angiography, *FAF* fundus autofluorescence, *HC* hydrocortisone, *HF* hyperfluorescent, *HAF* hyperautofluorescent, *OCT* optical coherence tomography, *OD* right eye, *OS* left eye, *RPE* retinal pigment epithelium, *SPH EQ* spherical equivalent of the manifest refraction, *SRF* subretinal fluid.

**Figure 1:**
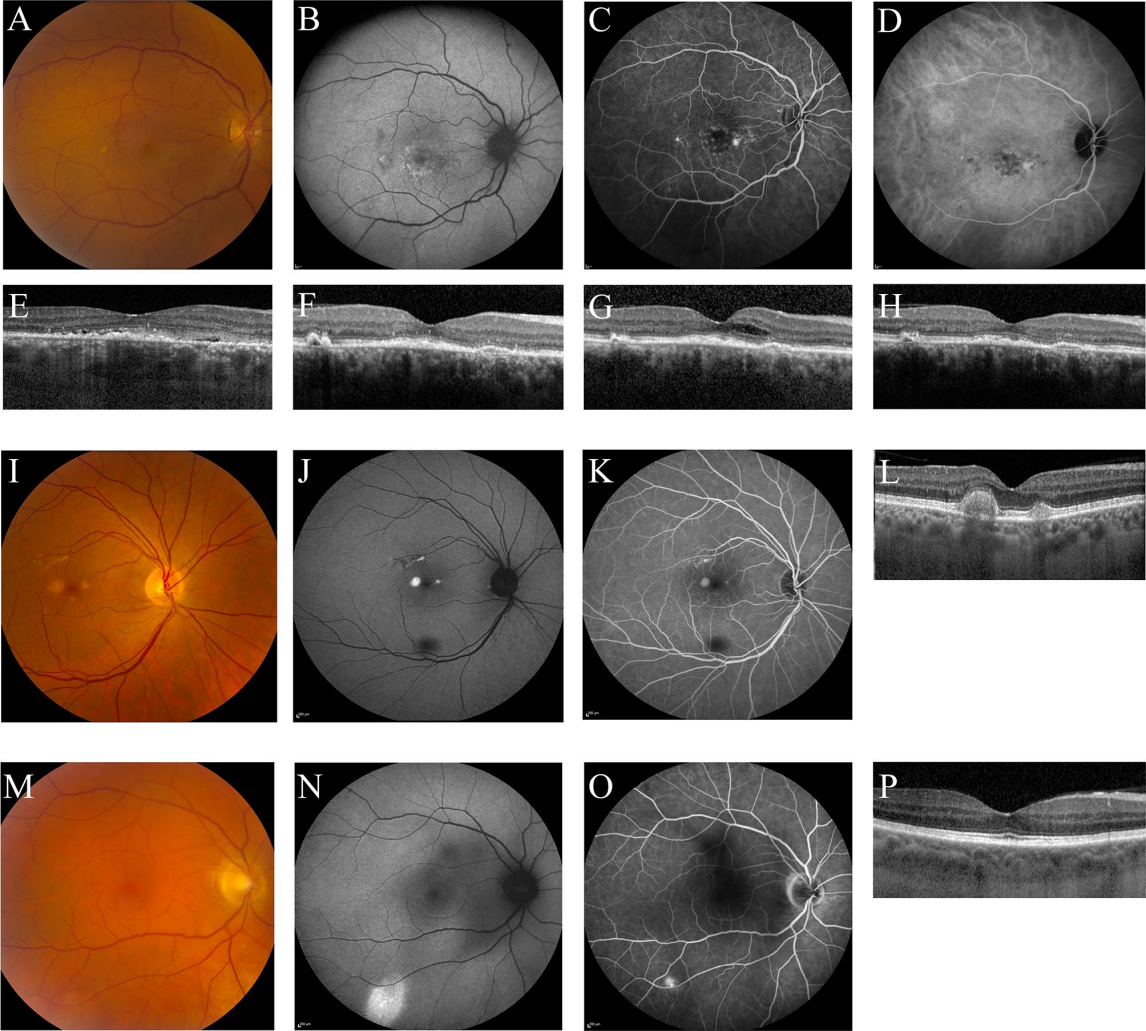
Multimodal imaging (**A**–**E**) of the right eye of a 66-year-old female patient with active Cushing’s syndrome. The optical coherence tomography (OCT) scan (**E**) revealed a small amount of subretinal fluid (SRF) with hyperreflective debris. Fundus autofluorescence (**B**) mainly showed hyperautofluorescent changes macularly. On the mid-phase fluorescein angiography (**C**) 2 ‘hot spots’ of leakage were observed, nasally and temporally to the fovea. Mid-phase indocyanine green angiography (**D**) at that time did not provide evidence for a neovascularization. In the left eye of this patient, no abnormalities were detected on multimodal imaging (not shown). As SRF persisted after 6 months of leuprorelin treatment for Cushing’s syndrome, half-dose photodynamic therapy was performed, after which the SRF almost completely resolved (**F**). However, 3 months later intraretinal fluid occurred (**G**), and intravitreal injections with bevacizumab were scheduled. After 3 initial injections once per month, a treat-and-extend protocol was used. Eight weeks after the last bevacizumab injection until to date, SRF and intraretinal fluid on OCT had disappeared (**H**). Unfortunately, visual acuity did not improve. Multimodal imaging of the right eye of a 63-year-old male with active Cushing’s syndrome (**I**–**M**). The foveal OCT scan (**L**) showed retinal pigment epithelium  alterations and parafoveal pseudovitelliform lesions. Corresponding hyperautofluorescent abnormalities were observed on fundus autofluorescence (**J**) and mid-phase fluorescein angiography (**K**). The abnormalities observed on multimodal imaging showed to be stable during follow-up, as can be seen on the OCT scan that was obtained at the last follow-up visit at 12 months after baseline (**M**). No abnormalities were observed in the left eye of this patient (imaging not shown). Multimodal imaging (**N**–**Q**) of the right eye of a 61-year-old male with active Cushing’s syndrome. No abnormalities were seen on the OCT scan (**Q**). Mid-phase fluorescein angiography (**P**) showed a ‘hot spot’ of focal leakage, with an eccentric location outside the vascular arcade, and on fundus autofluorescence (**O**) hyperautofluorescent changes were observed in the same area. Unfortunately, this area was outside the covering area of the OCT scan, so possible presence of SRF could not be evaluated. No abnormalities were observed in the left eye of this patient (imaging not shown).

In 3 out of the 11 patients CSC or CSC-like changes reminiscent of chronic CSC were observed (Fig. 1). In one of these patients, which had a history of grade 4 hypertensive retinopathy, SRF was observed on OCT in the right eye, accompanied with RPE alterations, outer retinal changes, and a flat irregular RPE detachment with midreflective accumulation beneath it, which raised the suspicion of a flat type 1 subretinal neovascularization below the RPE. In this patient, 2 parafoveal ‘hot spots’ of leakage were observed on FA, with corresponding hyperfluorescence on indocyanine green angiography, which was obtained for diagnostic purposes, and showed neither a clear neovascularization nor signs of polypoidal choroidal vasculopathy (aneurysmal type 1 neovascularization) (Fig. 1A–E). This patient was diagnosed with CSC and treated with half-dose photodynamic therapy (PDT), which initially resulted in a complete resolution of SRF (Fig. 1F). Half-dose PDT treatment was given 5 months after the start of leuproreline injections which was prescribed to treat the CS. Approximately 3 months after half-dose PDT, intraretinal fluid occurred (Fig. 1G), which resolved approximately 3 months after treatment with anti-vascular endothelial growth factor injections (Fig. 1H). Interestingly, no abnormalities were seen in the left eye of this patient on any of the imaging modalities (imaging not shown). The second patient showed RPE alterations and parafoveal pseudovitelliform lesions in the right eye with corresponding hyperfluorescent changes on FA and FAF (Fig. 1I–L). For this patient 2 more follow-up visits were scheduled 3 months and 12 months after the start of levoketoconazole as treatment for CS, during which no changes were observed (Fig. 1M). The third patient showed a single ‘hot spot’ of leakage on FA in the right eye with a corresponding hyperautofluorescent area on FAF (Fig. 1N–Q). This patient preferred not to receive follow-up for this study.

**Figure 2:**
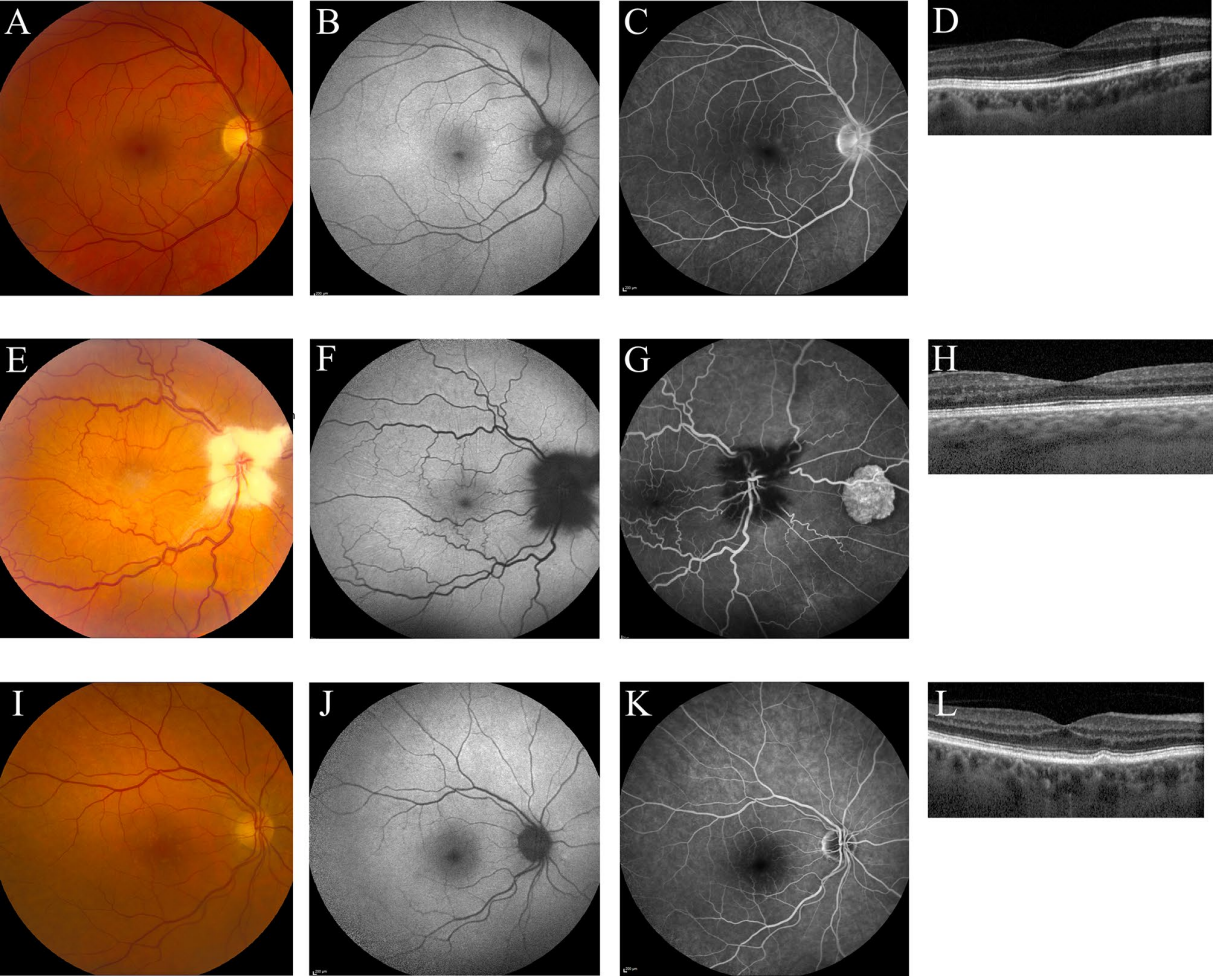
Multimodal imaging of ocular abnormalities not attributed to the spectrum of central serous chorioretinopathy. A 58-year-old male patient with Cushing’s syndrome due to a pituitary adenoma and a history of central retinal vein occlusion in the right eye (5 years ago) and grade 2 hypertensive retinopathy (3 years ago) showed mild parafoveal hyperfluorescent changes on fluorescein angiography (**C**). No abnormalities were observed on fundus photography (**A**), fundus autofluorescence imaging (**B**), and the foveal optical coherence tomography (OCT) scan (**D**). Furthermore, no abnormalities were found in the left eye on any of the imaging modalities (imaging not shown). The observed abnormalities in the right eye were stable after 1 year of follow-up after the first visit (imaging not shown). A 50-year-old male patient with Cushing’s syndrome due to a pituitary adenoma, who had a history of amblyopia in the right eye. Ophthalmological examination showed myelinated fibers which were present in both eyes (**E**–**G**). Furthermore, on fluorescein angiography (**G**) congenital hypertrophy of the retinal pigment epithelium was visible in the right eye. Due to amblyopia in the right eye, an OCT scan of sufficient quality could not be obtained. Therefore, an OCT of the left eye is shown, on which no abnormalities were observed (**H**). Multimodal imaging of the right eye a 75-year-old female with Cushing’s syndrome due to an adrenal adenoma, which showed drusen on fundus photography (**I**), with only very mild changes on fundus autofluorescence imaging (**J**), mid-phase fluorescein angiography (**K**), and the foveal OCT scan (**L**). These observations were similar in the left eye (imaging not shown).

In 8 out of the 11 patients, no abnormalities reminiscent of diseases within the CSC spectrum were found on multimodal imaging. One of the patients within this group was diagnosed elsewhere with central retinal vein occlusion in the right eye and grade 2 hypertensive retinopathy, 3–5 years before ophthalmological screening in the context of the current study. This patient showed RPE alterations and subtle hyperfluorescent changes on FA, which was considered to be characteristic of chronic arterial hypertension (Fig. 2A–D). These changes showed to be stable over time at a second visit within the current study. For 2 other patients within this group a second visit was scheduled as well, but also for these patients no changes were observed compared to the first visit (Table 2). Another patient showed myelinated nerve fibers and an epiretinal membrane in both eyes, together with congenital hypertrophy of the RPE in the right eye (Fig. 2E–H). A patient showing drusen formation in both eyes on OCT was previously diagnosed with dry age-related macular degeneration (Fig. 2I–L). Five patients within this group preferred not to receive follow-up for this study.

## Discussion

To the best of our knowledge, this is the first study in which complete ophthalmological examination has been performed in a consecutive series of CS patients with active disease. Ophthalmological changes could be detected in 3 of the 11 included patients without visual complaints. One of the CS patients was diagnosed with CSC based on unilateral SRF accumulation accompanied by corresponding hyperfluorescent changes on FA and indocyanine green angiography. This patient was initially successfully treated with half-dose PDT (which was performed 5 months after leuproreline injections were started to treat CS), which resulted in complete resolution of SRF. Three months after half-dose PDT, intraretinal fluid occurred, which was successfully treated with anti-vascular endothelial growth factor injections. Together with this intraretinal fluid a flat irregular RPE detachment with a midreflective accumulation beneath it was observed, suggesting that RPE detachments in patients with active CS could be rather fibrous than serous. It could, however, be hypothesized that the normalization of cortisol levels may also have contributed the successful treatment of the ocular pathology^6^. In 2 other patients hyperfluorescent changes on FA reminiscent of a ‘hot spot’ of leakage as typically seen in CSC patients could be observed, together with unilateral pseudovitelliform lesions in one of these subjects. Interestingly, posterior subcapsular cataract, a rare ocular manifestation of CS^19^, was not observed in this study.

CSC is a poorly understood chorioretinal disease, for which corticosteroid exposure has been found to be the most pronounced risk factor^9,12,20,21^. It has been hypothesized that this exposure only leads to characteristic chorioretinal abnormalities in patients with a certain disease susceptibility^22,23^. The results of our study confirm that endogenous hypercortisolism may indeed trigger the occurrence of ophthalmological changes within the CSC spectrum. As findings within this spectrum were only observed in the minority of the patients who developed extraordinary hypercortisolaemia as observed in CS, this study emphasizes that a local ocular susceptibility for developing CSC may play a role, increasing the risk of developing CSC upon exposure to corticosteroids^24^. This is supported by findings on multimodal imaging of CSC patients, which demonstrates that choroidal abnormalities on OCT (pachychoroid and pachyvessels) and indocyanine green angiography (choroidal stasis and hyperpermeability) frequently occur bilaterally, whereas actual accumulation of SRF and active leakage through the RPE on FA occur unilaterally in most cases^14,20,25,26^. Additionally, previous reports have found that even topical use of exogenous corticosteroids can trigger CSC^9,27^, indicating ‘hypersensitivity’ of some patients to develop CSC in response to modest amounts of exogenous corticosteroids.

Our findings are partially in line with the findings from a cross-sectional study in which 11 patients with active CS and 12 healthy controls were included, and in which bilateral macular changes characteristic for (subclinical) CSC on OCT were seen in 1 patient^17^. In contrast with our study, a significantly increased choroidal thickness compared to healthy subjects could be detected in these 11 patients^17^. Moreover, episodes of hypercortisolaemia in CS patients have previously been linked to CSC by Bouzas et al., who included 60 CS patients, out of whom 3 cases developed hypercortisolaemia-dependant episodes of CSC. In contrast with our study, this study also included patients that had no active disease, which might explain the fact that we have found a higher percentage of patients with abnormalities within the CSC spectrum^8^.

In our study, we only included patients with active CS and performed multimodal ophthalmological imaging in all patients, which is a particular strength of the study. Additionally, 5 of the 11 patients visited the outpatient clinic at least 2 times. There are several limitations in our study. Importantly, our sample size is limited, which is mostly due to the rare incidence of CS. Another limitation of our study might be that 6 patients were already receiving treatment at the moment of ophthalmological phenotyping in an attempt to control endogenous cortisol production. Due to logistics with scheduling visits to the outpatient clinics, patients were on medical pre-treatment for varying periods of time. Because of the seriousness of the disease, it was considered not ethical to withhold this treatment before the ophthalmological examinations. However, only in 3 patients this treatment was prescribed for more than 7 days, and in all patients biochemical analysis still showed hypercortisolism at the time of ophthalmological evaluation.. This is in line with a previous study showing that cortisol lowering agents most often do not lead to complete normalization of hypercortisolaemia^28^. We therefore assume that it is unlikely that the medical pretreatment has influenced the outcome of our study. Albeit, if it had any effect, it could only have lowered the incidence of CSC(-like) abnormalities found in this study. Furthermore, our study included CS patients with a mixed origin of hypercortisolaemia. However, we expect that this will not have influenced the outcome of our study, since hypercortisolaemia, which is considered to be the most important risk factor for developing CSC, was present in all included patients. Notwithstanding, although there is a common trunk of hypercortisolism in CS, it is very interesting to better understand the origin of CS on CSC by studying a larger cohort – probably multicenter. This would also allow for a more comprehensive analysis of risk factors associated with CSC such as gender, or manifestations of CS such as neuropsychiatric symptoms or biochemical (hormonal) abnormalities such as ACTH^29^.

The relationship between CS and CSC is relatively well-known^12^. CSC is a relatively rare disease, for which an incidence of 9.9 per 100,000 for males and 1.7 per 100,000 for females has been described in a retrospective cohort study^30^. Of note, our study included 4 male CS patients and 7 female CS patients and we observed CSC(-like) abnormalities in 2 males and 1 female, despite the fact that CS occurs 5 times more often in females^29^. The incidence of CSC(-like) ophthalmological changes, which occurred in 3 out of the 11 CS patients in our study, may suggest that (subclinical) CSC is more likely to occur in CS patients compared to the general population. Furthermore, CSC has previously been shown to occasionally be the presenting symptom of CS^6^. Moreover, CS surgery has been observed to result in complete and persistent resolution of SRF^6^. However, in a systematic cross-sectional study that recently evaluated 86 CSC patients for presence of CS, we previously did not find any cases of (subclinical) CS^31^. Collectively, it might be of clinical importance to screen CS patients for pathology within the CSC spectrum on a regular basis. In the current study, we found active CSC with unilateral SRF in 1 patient without visual complaints, who required treatment. Moreover, abnormalities reminiscent of (subclinical) CSC were observed in 2 other patients. Patients with active CS should therefore be actively questioned about the presence of ophthalmological symptoms and referred to an ophthalmologist if such symptoms are present. Further studies are needed to assess whether all patients presenting with CS should be referred for an ophthalmological screening.

In conclusion, findings suggestive of (subclinical) CSC and other ocular fundus abnormalities were detected in a noteworthy percentage of patients with active CS without visual complaints. Ophthalmological screening including multimodal imaging may be indicated in recently diagnosed patients with (active) CS. However, additional studies on the prevalence of subclinical CSC in CS and the natural course of these abnormalities (also after CS treatment) are required to determine whether standard ophthalmological screening of newly diagnosed CS patients should be incorporated in the clinical work-up of patients diagnosed with CS.
